# Therapy options in deep sternal wound infection: Sternal plating versus muscle flap

**DOI:** 10.1371/journal.pone.0180024

**Published:** 2017-06-30

**Authors:** Martin Grapow, Martin Haug, Chistopher Tschung, Bernhard Winkler, Prerana Banerjee, Paul Philipp Heinisch, Jens Fassl, Oliver Reuthebuch, Friedrich Eckstein

**Affiliations:** 1 Department of Cardiac Surgery, University Hospital, Basel, Switzerland; 2 Department of Plastic, Reconstructive, Aesthetic, and Hand Surgery, University Hospital, Basel, Switzerland; 3 Department of Cardiovascular Surgery, Inselspital, University Hospital, Bern, Switzerland; 4 Department of Anaesthesiology, University Hospital, Basel, Switzerland; University of Insubria, ITALY

## Abstract

**Background:**

Management of deep sternal wound infection (DSWI) in cardiac surgical patients still remains challenging. A variety of treatment strategies has been described. Aim of this cohort study was to analyse two different treatment strategies for DSWI: titanium sternal plating system (TSFS) and muscle flap coverage (MFC).

**Methods:**

Between January 2007 and December 2011, from 3122 patients undergoing cardiac surgery 42 were identified with DSWI and treated with one of the above mentioned strategies. In-hospital data were collected, follow-up performed by telephone and assessment of Quality of Life (QoL) using the SF-12 Health Survey Questionnaire.

**Results:**

20 patients with deep sternal wound infection were stabilized with TSFS and 22 patients treated with MFC. Preoperative demographics and risk factors did not reveal any significant differences. Patients treated with TSFS had a significantly shorter operation time (p<0.05) and shorter hospitalization (p<0.05). A tendency towards lower mortality rate (p = n.s.) and less re-interventions were also noted (plating 0.6 vs. flap 1.17 per patient, n.s.). Quality of Life in the TSFS group for the physical-summary-score was significantly elevated compared to the MFC group (p<0.05). Relating to chest stability and cosmetic result the treatment with TSFS showed superior results, but the usage of MFC gave the patients more freedom in breathing and less chest pain.

**Conclusion:**

Our results demonstrate that the use of TSFS is a feasible and safe alternative in DSWI. However, MFC remains an absolutely essential option for complicated DSWI since the amount of perfused tissue can be the key for infection control.

## Introduction

Conventional medium sternotomy is the most common access to the heart and mediastinum for cardiac surgeons since Julian et al. re-introduced Milton's operation for this access in 1957 [1]. Deep Sternal Wound Infection (DSWI) with sternal dehiscence after cardiac surgery is nowadays a rare but highly feared complication associated with considerable morbidity and mortality [2]. Longer duration of hospitalization, expansive antibiotic treatments, reinterventions and significantly increased costs to healthcare systems are relevant consequences [3–7]. Several authors have reported about 16 up to 30 additional hospital days compared to patients with uncomplicated postoperative courses [8–11]. Upton et al. showed an up to 2-fold increase of cost per patient [6], Lee et al. described an additional cost of $ 500'000 per incident of poststernotomy mediastinitis [5].

A variety of treatment concepts like extended debridement, open dressing-changes with secondary wound healing, Robiscek-wire closure, irrigation-suction drainage, closed drainage with multiple redon catheters at high negative pressure and others have been introduced in the past [12–19], but failure rates remained high. Thorough debridement, culture-directed antibiotics and the use of vacuum-assisted therapy (VAC) significantly decreased mortality and morbidity and is nowadays standard as first-line intervention for DSWI [12].

The transposition of a vascularized muscle flap as final therapy is considered to be the gold standard due to its tremendous potential of infection control. Application of a pectoralis major muscle flap coverage (MFC) in this setting was first described by Jurkiewics in the 80’s [20]. Several authors considered using MFC as the primary choice for wound closure in poststernotomy mediastinitis [15–17,21,22]. Besides the pectoralis major muscle flap a lot of different techniques like advancement, rotational or turnover flaps but also tissue sources such as rectus abdominis, latissimus dorsi muscle, and omentum plombing have been described in the reconstruction of dehiscent, infected sternotomy sites [21–23].

In 2005 Cicilioni et al. introduced sternal reconstruction with a transverse titanium sternal plating system (TSFS) in conjunction with a bilateral pectoralis advancement flap in DSWI [24]. The promising results were in line with later observations supporting this method as an alternative treatment option in the setting of infection, sternal dehiscence [25,26].

However, a general lack of consensus regarding the optimal treatment regimens and reconstructive techniques for this complication still exists [27]. The aim of this study was to perform a retrospective analysis in comparing both treatment, TSFS vs. MFC for the treatment of post-cardia DSWI, with special focus on Quality of Life and outcome to respect the most valid therapy suitable for this specific patients’ cohort.

## Methods

### Patient collective

Our retrospective analysis included all patients presenting with post-sternotomy DSWI, who underwent either sternal plating or muscular flap reconstruction from January 2007 to December 2011. An observational design was used in this analysis, conforming to the STROBE statement and checklist [28]. From a total of 3122 patients undergoing cardiac surgical operations with sternotomy at our institution. Overall 73 (2.27%) patients were identified having DSWI and of those 42 (1.35%) patients were treated exclusively with either TSFS or MFC. In this patient collective, the initially received cardiac procedures are listed in S1 Table. After diagnosis of DSWI and its pathogen all patients in both groups received wound preconditioning using VAC-therapy and antibiotics (100%) [29,30]. The most common isolated pathogen (S2 Table) was coagulase-negative staphylococcus in 50% of the cases (n = 24), staphylococcus aureus in 15% (n = 7) and enterobacteriaceae in 8% (n = 4). There were two cases of methicillin-resistant Staphylococcus aureus (MRSA). Additional information is provided in S2 Table.

**Fig 1:**
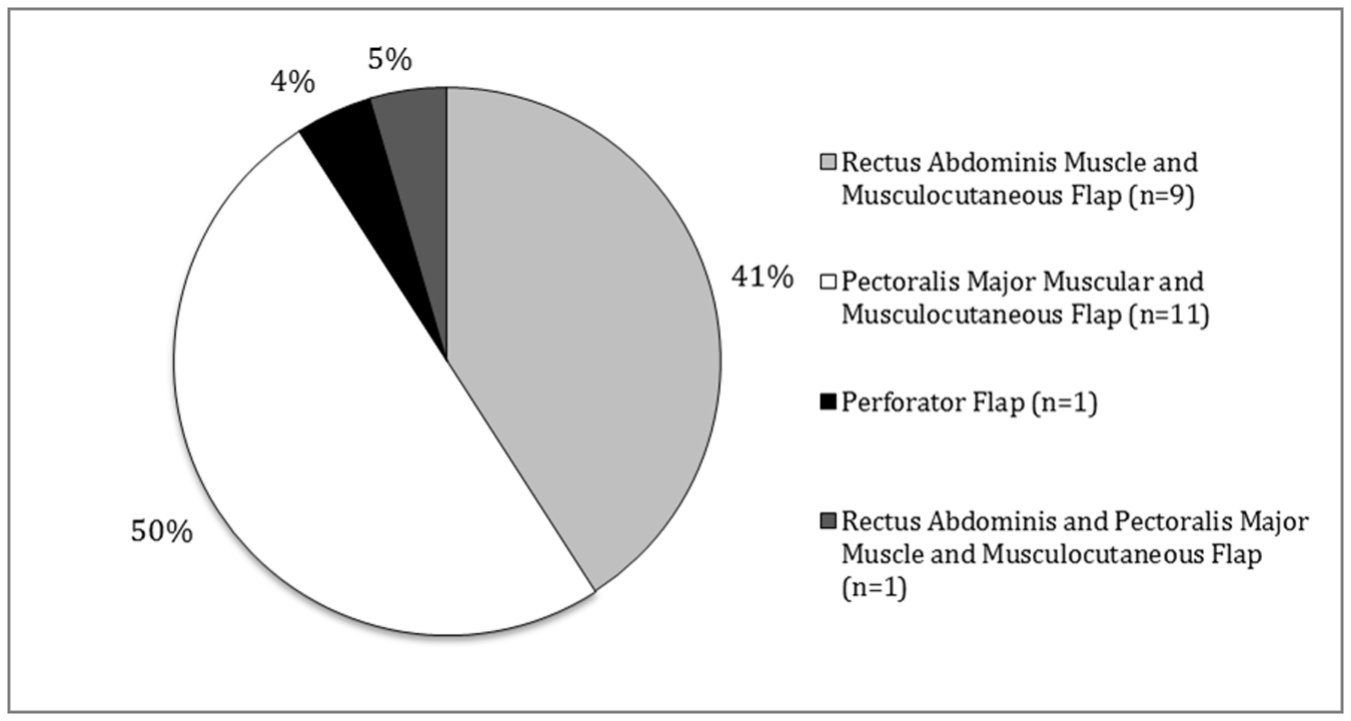
Muscle Flaps used in DSWI.

A total of 20 patients received a stabilization with TSFS (15 males and 5 females). Furthermore, 22 patients (15 males and 7 females) were treated by MFC (Fig 1): rectus abdominis muscle-musculocutaneous flap (n = 9), pectoralis major muscular-musculocutaneous flap (n = 11), perforator flap (n = 1) and rectus abdominis—pectoralis major muscle—musculocutaneous flap (n = 1). The choice was made according to the surgeons preference and experience with the techniques.

The remaining 31 patients were treated with direct closure or the combination of VAC with delayed closure using wires in standard-fashion or the Robiscek-approach and were not part of our investigation.

Therefore, the cohort in this study represents a limited selection/population, containing patients with poor bone quality, failed first attempt or even resected sternum. The database was generated from patient charts of the University Hospital Basel, Switzerland.

### Definition criteria

The definition of DSWI is according to the guidelines of the Centres for Disease Control and Prevention [31]: The definition of DSWI requires the presence of at least one of the following criteria: 1) an organism isolated from culture of mediastinal tissue or fluid; 2) evidence of mediastinitis seen during operation; or 3) presence of either chest pain, sternal instability, or fever (>38°C), and either purulent discharge from the mediastinum, isolation of an organism present in a blood culture, or culture of drainage of the mediastinal area.

After diagnosis of DSWI and its pathogen all patients in both groups received wound preconditioning using Vac-therapy and i.v. antibiotics (100%) until the mediastinum was properly cleaned and macroscopically without any signs of infection [29,30].

### SF-12 questionnaire

The local ethics committee of the university hospital Basel approved this study. Written informed consent was obtained from all participating patients prior to the intervention and for a Quality of Life assessment with the standardized SF-12 Quality of Life Questionnaire Interview Form (Hogrefe Webedition). The physical and mental health sum scales were computed using the scores of twelve questions and range from 0 to 100, where a zero score indicates the lowest level of health measured by the scales and 100 indicates the highest level of health. Both physical and mental sum scores were computed that they compare a national norm score to have means of 50.0 and a standard deviations of 10.0 [32].

Besides the standardized SF-12 Quality of Life Questionnaire, six specific questions were included. Frequency of chest pain, constricted/captivated feeling and problems in breathing were asked, with four possible answers: from 1 to 4, score 1 indicates the highest level and 4 indicates the lowest level of having problems. Satisfactory of chest stability and cosmetic result were analyzed: score 1 indicates the lowest and 4 the highest level of satisfactory. The feeling of foreign body was asked, 1 for "yes" and 0 for "no". All patients were contacted and interviewed by phone. For better viewing and understanding purposes the answers have been transformed in percentages: 0%, 33%, 66%, 100%.

### Statistical analysis

The SF-12 questionnaire is a 12-item short health survey for self-administration as well as scripts for personal interviews. We used the standard version for telephone interview provided by Hogrefe Testsystem (Hogrefe Publishing Corp.) which was completed by our questionnaire focused on relevant questions. Continuous variables were expressed as mean with standard deviation, median and range, whereas categorical data were expressed as percentages.

For continuous variables, values were compared using the Mann Whitney U-Test. Differences in proportion were compared using Fisher’s exact test. A p value of less than 0.05 was considered statistically significant. All statistical analysis was performed using IBM SPSS Statistics 20 for Windows.

## Results

### Demographic data

Preoperative patient characteristics revealed no significant differences, although patients receiving a MFC presented with an increased EuroScore, which was mainly driven by a reduced left ventricular ejection fraction (LVEF) and a frequent history of myocardial infarction (Table 1). Patients undergoing TSFS had a higher body mass index (BMI) and a more frequent history of cerebral vascular insult (CVI).

**Table 1:** Preoperative patient characteristics for each treatment group.

	TSFS (n = 20)	MFC (n = 22)	p Value
**Age at Operation**	70.5 (40–83), 70±9.7	73 (54–84), 70.8±8.5	0.74
**Male Gender**	15 (75.0)	15 (68)	0.74
**EuroScore additive**	7 (3–12), 7.8±2.9	8 (5–15), 8.8±2.9	0.32
**EuroScore logistic**	7.04 (2.56–29.92), 12.0±9.0	9.78 (3.44–52.25), 14.9±12.8	0.32
**LVEF, %**	59 (30–89), 53.4±15.7	50 (20–80), 48.4±16.9	0.39
**Body Mass Index, kg/m2**	31.5 (25–50), 33.7±7.6	31 (20–44), 30.6±6.3	0.23
**Diabetes mellitus**	12 (45.0)	11 (50.00)	0.77
**Chronic Obstructive Pulmonary Disease**	5 (25.0)	2 (9.09)	0.23
**Hypertension**	16 (80.0)	20 (90.91)	0.40
**Peripheral Vascular Disease**	4 (20.0)	3 (13.64)	0.69
**Active smoker or stopped <3 months**	3 (15.0)	3 (13.64)	1.00
**Steroids**	2 (10.0)	1 (4.55)	0.60
**HO Stroke/Transient Ischemic Attack**	6 (25.0)	1 (4.55)	0.087
**HO Myocardial Infarction**	9 (45.0)	17 (77.27)	0.055
**Osteoporosis**	2 (10.0)	2 (9.09)	1.00
**Re-Do surgery**	0 (0.00)	2 (9.09)	0.49

Data are presented as Median and Range with Mean ± standard deviation or absolute value and percentage (%). HO = History of. LVEF = Left ventricular ejection fraction

### Time period between cardiac surgery and final closure

Postoperative data analyses revealed significantly more first attempts of operative sternal re-stabilization in the MFC group (86.6%vs. 35.0%, p = 0.001). Statistically significant differences were also found in the number of VAC-dressing changes before final treatment for sternal dehiscence (MFC 3.7±1.2 versus 2.7±1.5 per patient, p = 0.012). A tendency towards longer ICU stays with a mean of 14.2 days versus 7.5 days and more resuscitation in the MFS group with 36.36% compared to only 15% in the TSFS group were evident, although being not significant (Table 2).

**Table 2:** Intra- and postoperative patient data.

	TSFS (n = 20)	MCF(n = 22)	p
Postoperative ICU Stay (d)	4.5 (2–27), 7.5±7.3	5 (2–69), 14.2±18.4	0.22
Cardiac Operation Time (min)	214.5 (134–462), 232.4±70.2	268 (115–415), 258.7±75.5	0.10
Emergency Operation	6 (30.0)	2 (9.09)	0.12
Pneumonia	12 (60.0)	11 (50.00)	0.55
Post-OP Delir	14 (70.0)	10 (45.45)	0.13
DSWI	20 (100)	22 (100.00)	1.00
Post-OP Bleeding	2 (10.0)	2 (22.73)	0.41
Seroma Formation	7 (35.0)	6 (27.27)	0.35
Tracheotomy	1 (0.05)	4 (18.18)	0.19
VAC usage in n patients	20 (100)	22 (100.00)	1.00
Number of VAC changes	3 (1–6), 2.7±1.5	4 (1–6), 3.7±1.2	0.012
Sternum Revision	7 (35.0)	19 (86.36)	0.001
Resuscitation	3 (15.0)	8 (36.36)	0.166

Data are presented as Median and Range with Mean ± standard deviation or absolute value and percentage (%). ICU = Intensive care unit. VAC = Vacuum-assisted closure

### Final closure and further course

Operation time for TSFS was significantly shorter compared to MFC (138.8±25.8 h vs. 184.3±75.9 h; p = 0.009) the postoperative ICU stay was comparable, the period from final reconstruction to hospital discharge was significantly shorter in the TSFS group (18.1±20.6 d vs. 38.9±39.3 d; p = 0.025).

Though statistically not significant, patients treated with TSFS had a tendency towards fewer secondary operative interventions (bleeding, seroma, necrosis, recurrent infection, hardware removal) after final reconstruction (0.6 ±1.1 vs. 1.2±1.9 per patient, p = 0.24) and a lower mortality rate (Table 3). In the TSFS group sternal plates were removed in one case due to seroma formation four months after surgery. There was no sign of infection and due to sternal stability the plates could be removed without an alternative stabilization.

**Table 3:** Patient data after Index therapy.

	TSFS (n = 20)	MFC (n = 22)	p
Prolonged ICU Stay >2d, Mean Stay at ICU (d)	25.0%, 2.9	22.73%, 3.18	1.000
Post-Op ICU Stay (d)	0 (0–20), 2.9	1.5 (0–37), 3.2	0.93
Operation time (min)	134.5 (98–180), 138.8±25.8	189 (38–297), 184.3±75.9	0.009
Time Difference (d) Heart Surgery to Final Reconstruction	23.5 (7–37), 23.5±7.9	42 (19–65), 41±14.4	<0.001
Days to Discharge	12 (1–91), 18.1±20.6	19.5 (8–137), 38.9±39.3	0.025
Seroma Formation	4 (20.0)	6 (27.27)	0.723
Post Op Bleeding	2 (10.0)	4 (18.1)	0.661
No. of Post Op Interventions, total	12; 0 (0–3), 0.6±1.1	28; 1 (0–7), 1.2±1.9	0.249
Hardware removal	2	0	0.22
30 day Mortality	2	2	0.187
1-year mortality	3	6	0.46
Death of Patient at Date of Follow Up	3 (15.0)	9 (40.9)	0.091

Data are presented as Median and Range with Mean ± standard deviation or absolute value and percentage (%). HO = History of. ICU = Intensive care unit. Postoperative Interventions due to bleeding, recurrent infection, seroma formation, resection of necrotic tissue, hardware removal. Hardware removal due to infection and pain.

Thirty-day mortality was 9.5%, 1-year-mortality was 21.4% and total all-cause-mortality after a mean follow-up of 33.42 months was 28.3% for the entire cohort. Overall mortality for the TSFS-group was 10% for 30 days, 15% for 1 year and 15% with a mean follow-up of 34.6 months. The MFC-group showed a 30-day mortality of 9.0%, 27% and 40% with a mean follow-up of 23.6 months. Six patients died in the MFC group, and three patients in the TSFS group within one year after final reconstruction, most of them due to multi organ failure (S3 Table).

### SF-12 questionnaire

In the TSFS group, 14 patients agreed to answer the SF-12 Quality of Life questionnaire in a 4 weeks range: 1 patient left the country and was lost to follow-up. Two patients got TSFS explanted at time of follow up and 3 patients died during time of follow-up. Only 8 Patients agreed to answer the questionnaire in the MCF group: Two patients were lost to follow-up. Another patient suffered from severe dementia and was unable to answer the questionnaire. In total 9 patients have died during the follow-up period, six patients within 1 year and 3 more patients beyond 1 year after final surgery. Two patients did not agree to participate.

**Fig 2:**
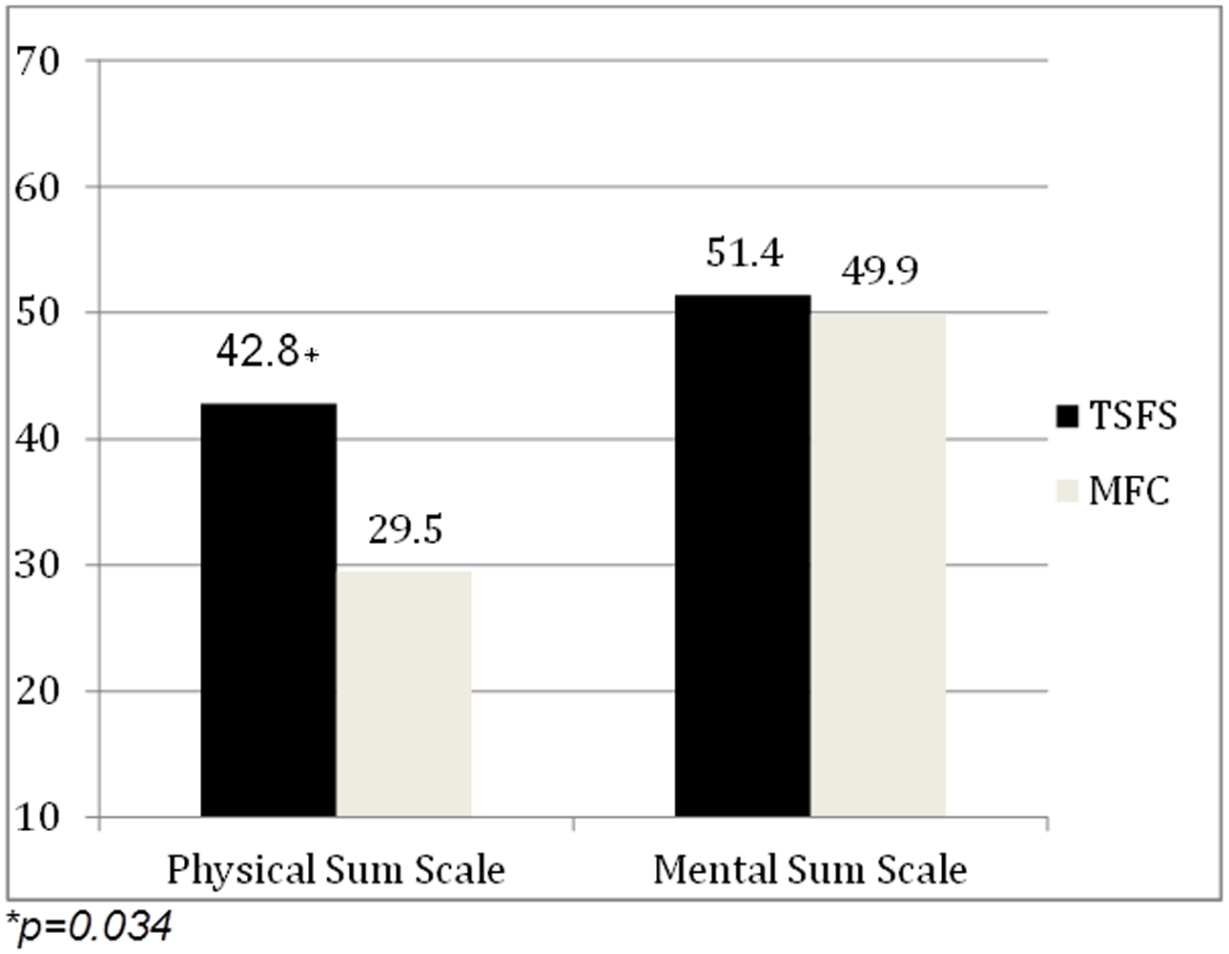
Results of the SF-12 questionnaire.

The standardized SF-12 questionnaire compared two aspects in quality of life on the Norm Scale Calculator, the physical sum scale revealed a statistically significant better physical quality of life for patients in the TSFS group (42.8±8.6 vs. 29.5±10.7 p = 0.034), (Fig 2) and (Table 4). The mental sum scale was similar between the groups. Further information regarding preoperative and postoperative patient characteristics for each treatment group in the SF12 questionnaire are presented tin S4 and S5 Tables.

**Table 4:** SF-12 questionnaire-results.

	TSFS (n = 14)	MFC (n = 8)	p-value
Physical Sum Scale	44.7 (27.7–56.2), 42.8±8.6	32.7 (21.6–48.8), 29.5±10.7	0.034
Mental Sum Scale	56.2 (29.0–64.1), 51.4±11.1	47.8 (24.7–64.8), 49.9±13.2±13.2	0.375

Data are presented as Median and Range, Mean± standard deviation. P Value has been calculated by Mann-Whitney U test.

### Specific questionnaire

**Fig 3:**
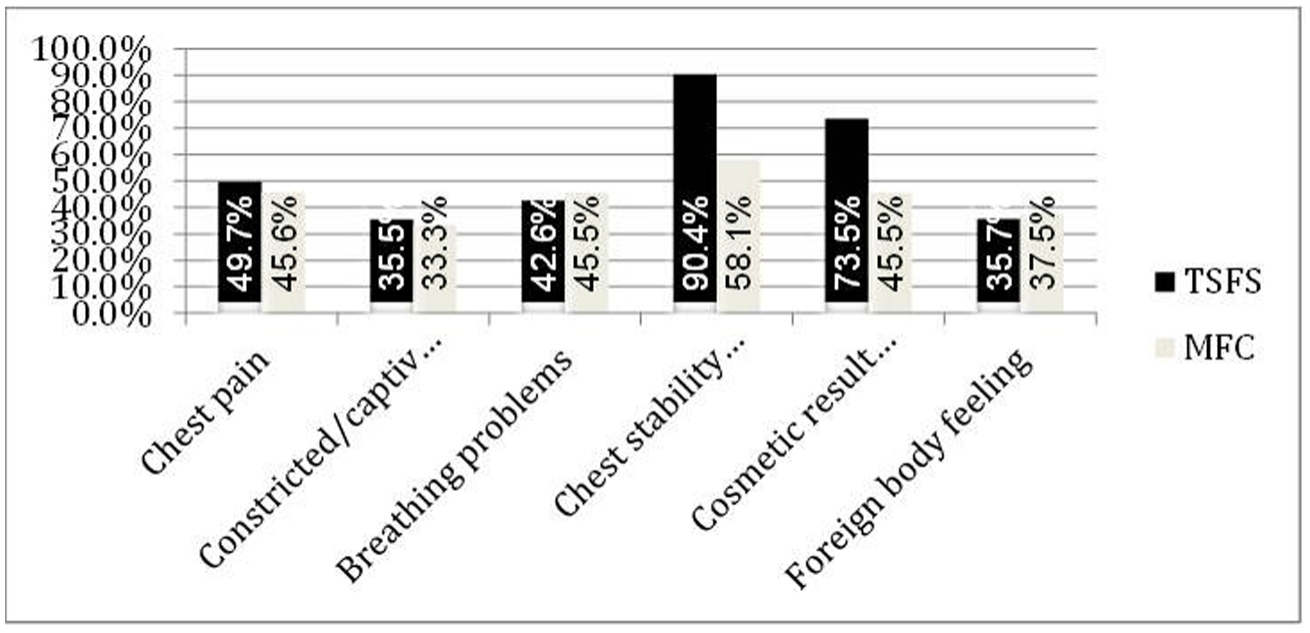
Specific questionnaire.

For those six additional questions no significant differences were obtained (Fig 3). Chest pain was seen more frequently in TSFS patients and satisfaction with the cosmetic result combined with the sensation of a stable chest was less likely in MFC patients. Specific patient data after definite therapy in SF-12 group are presented in S6 Table.

## Discussion

The application of a transverse titanium sternal plating system was first described by Cicilioni et al. in 2005 [24]. Since then evidence has accumulated that the approach of using titanium plate osteosynthesis in a preconditioned and preserved sternal surrounding offered by modern VAC therapy has the potential to become an alternative in the therapy of complex DSWI [12,16,26]. The goal of this study was to analyse the effect of different treatment options in DSWI, TSFS vs. MFC, on quality of life and outcome.

Despite numerous advancements in perioperative care, 30-day mortality was 9.5%, 1-year-mortality was 21.4% and total all-cause-mortality after a mean follow-up of 33.42 months was 28.3% for the entire cohort. The results are comparable to the data published by Milano et al [33] analysing long-term outcome of CABG-patients with mediastinitis, a mortality of 28.6% was demonstrated at 1.5 years. Another study by the Leipzig group shows an in-hospital-mortality of 21.6%. In this study in-hospital-mortality was significantly lower (17.1%) in patients undergoing isolated CABG, but increased in patients undergoing other procedures (43.5%) [34]. Our patients underwent in 64.2% isolated CABG, the remaining 35.8% combination surgery.

Although the preoperative demographic data were almost similar between the groups, they differed in a few but important aspects after cardiac surgery. A tendency towards longer ICU stays and more resuscitation in the MFS group were evident, although being not significant. However, secondary closure attempts (after initial cardiac operation) with wires have been performed significantly more often in the MFC group in 86.36% versus 35% in the TSFS group. Gummert et al. reported increased hospital mortality in patients requiring more than one operation to treat the infection [34]. Furthermore, VAC-dressing-changes were carried out significantly more often for MFC patients as compared to TSFS patients.

This again had major impact on the time interval cardiac-surgery-to-final-sternal-reconstruction, which was consecutively delayed for the MFC cohort. One possible explaining factor is the patient-referral to our Division of Plastic and Reconstructive Surgery accompanied by a prolonged operative planning period, whereas TSFS patients were scheduled and operated by cardiac surgeons. Karra et al. conducted a multivariate analysis of risk factors for 1-year mortality after postoperative mediastinitis. The group concluded that a delay in sternal closure to be a major highly significant independent predictor with a hazard ratio of 6.27 [35]. In contrast to a large 15-year review by Baillot et al. who reported a decreased perioperative mortality treated with VAC followed by TSFS in 92 patients compared with muscle flaps [12].

In the current study 30-day mortality was almost identical between the groups in our cohort. There was only a tendency towards an increased long-term mortality. Patients treated with TSFS were better by trend with a total all-cause-mortality of 15% after a mean follow-up of 3 years compared to 40.9% after a mean follow-up of 25.9 months. Patients supplied with TSFS were discharged significantly earlier, while time of intubation and time on ICU were similar between the groups. Although statistically not significant operative interventions due to postoperative bleeding, seroma formation, recurrent infection, resection of necrotic tissue or hardware removal were performed by trend less often in the TSFS group. This again might reflect the different health condition in both groups before final sternal treatment. All over 5 out of 20 patients had to be reoperated in our cohort which is similar to the data published by Schols et al. [16]. They presented their results in 22 patients, who were supplied by sternal plates and pectoralis mayor advancement flaps, too. Although 15 patients showed an uneventful postoperative course, 7 patients complained about fistulas and wound dehiscence, 4 of them presented with a recurrence of infection.

To our knowledge this is the first analysis in which outcome and QoL are directly compared between patients treated with the TSFS and MFC. Our results of the SF-12 Quality of Life Questionnaire concerning the physical summary scale show, that patients treated with the TSFS have a significantly better quality of life (p = 0.03).

Interestingly the physical situation did not affect the psychological status, i.e. the mental summary scale, which did not reveal any relevant differences.

Plass et al. investigated preoperative and postoperative pulmonary function test on 41 patients treated with the TSFS in their study. All of the patients showed no worsening in respiratory function [26]. In our questionnaire only 2 of 14 (14%) patients complained of “often” having problems in breathing, 5 of 14 (36%) “sometimes”. Whereas in the MFC group 1 of 8 (13%) complained of “often”, and 4 of 8 (50%) “sometimes” having problems in breathing. Patients with a TSFS reported more often about pain, but on the other hand they were more satisfied with the cosmetic result and with the achieved stability of the chest. There was no significant difference found on the results of the questionnaire.

We acknowledge some limitations of our study. Due to the low incidence of DSWI the number of investigated patients in our study is small. Patients included were operated in a five-year-period between 2007 to 2011 in order to have a similar spectrum of indication, therapeutical options and of course a reasonable number of patients. Another limitation is that this study is a retrospective analysis. Although demographic data for both patient groups were similar before cardiac surgery both groups developed differently in the period between cardiac surgery and final sternal stabilization in favour of the TSFS group. Best evidence is guaranteed by performing a prospective randomized controlled multi-center study.

The evaluation of the SF-12 Quality of Life questionnaire has also to be done with caution, since the problem of confounding factors cannot totally be ruled out. The questionnaire is a subjective test and does not provide hard facts.

Several advantages are assumed for TSFS compared to MFC closure with proposed early postoperative extubation, decreased sternal pain, improved comfort, decreased mediastinal hernia and paradoxical chest wall breathing [25,26]. The results demonstrate, that the use of TSFS is a potential and safe option in DSWI with respect to the limitations of this study.

Besides less complication, shorter hospital stays and lower mortality rate. In the long-term follow-up a better quality of life on the physical norm scale was found. However, MFC remains an absolutely essential therapy option for complicated DSWI since the amount of perfused tissue can be the key for infection control. Nevertheless, the usage of TSFS should be evaluated for every patient. Through the use of the TSFS the anatomical, biomechanical and physiological function of the chest is maintained, furthermore it is the less invasive therapy option.

## Supporting information

S1 Table Cardiac procedures. (DOCX)

S2 Table Isolated pathogens from wound cultures. (DOCX)

S3 Table Cause of death and 1-year mortality. (DOCX)

S4 Table Preoperative patient characteristics for each treatment group in SF12 group. (DOCX)

S5 Table Intra- and postoperative patient data in SF-12 group. (DOCX)

S6 Table Patient data after definite therapy in SF-12 group. (DOCX)
