# Supplementary material for: Therapy options in deep sternal wound infection: Sternal plating versus muscle flap
Source: PLoS One. 2017 Jun 30;12(6):e0180024. doi: 10.1371/journal.pone.0180024 (PMC5493354; doi:10.1371/journal.pone.0180024)
Supplement: S1 Table — (DOCX) [file pone.0180024.s001.docx]

*Suppl. Table 1*

*Cardiac procedures*

|  | TSFS (n=20) | MFC (n=22) |
| --- | --- | --- |
| Coronary artery bypass | 12 | 15 |
| Aortic valve repair | 2 | 1 |
| Coronary artery bypass + Aortic valve repair | 2 | 2 |
| Mitral valve repair + tricuspid valve repair | 1 | 0 |
| Mitral valve repair | 1 | 2 |
| Coronary artery bypass + mitral valve repair | 0 | 2 |
| Aortic valve repair + mitral valve repair | 1 | 0 |
| Coronary artery bypass + tricuspid valve repair | 1 | 0 |
