# Supplementary material for: Therapy options in deep sternal wound infection: Sternal plating versus muscle flap
Source: PLoS One. 2017 Jun 30;12(6):e0180024. doi: 10.1371/journal.pone.0180024 (PMC5493354; doi:10.1371/journal.pone.0180024)
Supplement: S2 Table — (DOCX) [file pone.0180024.s002.docx]

*Suppl. Table 2*

*Isolated pathogens from wound cultures*

|  | Absolute number (Percentage) |
| --- | --- |
| Coagulase-Negative Staphylococcus | 24 (50) |
| Staphylococcus aureus | 7 (15) |
| Enterobacteriaceae | 4 (8) |
| MRSA | 2 (4) |
| Propionibacterium | 2 (4) |
| Proteus vulgaris | 2 (4) |
| Klebsiella | 2 (4) |
| Pseudomonas aeruginosa | 2 (4) |
| Corynebacterium | 1 (2) |
| Escherichia coli | 1 (2) |
| Enterococcus faecalis | 1 (2) |
