# Supplementary material for: Therapy options in deep sternal wound infection: Sternal plating versus muscle flap
Source: PLoS One. 2017 Jun 30;12(6):e0180024. doi: 10.1371/journal.pone.0180024 (PMC5493354; doi:10.1371/journal.pone.0180024)
Supplement: S3 Table — (DOCX) [file pone.0180024.s003.docx]

Suppl. Table 3

*Cause of Death, 1-year mortality*

| Patient No. | Age | Gender | Survival after Plating/Flap (d) | Cause of Death |
| --- | --- | --- | --- | --- |
| MFC 1 | 73 | m | 17 | Multi organ failure |
| MFC 2 | 55 | m | 38 | Lung emboli |
| MFC 3 | 73 | m | 132 | Sepsis/Multi organ failure |
| MFC 4 | 75 | m | 17 | Sepsis/Multi organ failure |
| MFC 5 | 76 | m | 68 | Multi organ failure |
| MFC 6 | 66 | m | 110 | Multi organ failure |
| TSFS 1 | 63 | f | 48 | Sepsis |
| TSFS 2 | 70 | f | 21 | Multi organ failure |
| TSFS 3 | 76 | m | 12 | Sudden cardiac death |
