# Supplementary material for: Therapy options in deep sternal wound infection: Sternal plating versus muscle flap
Source: PLoS One. 2017 Jun 30;12(6):e0180024. doi: 10.1371/journal.pone.0180024 (PMC5493354; doi:10.1371/journal.pone.0180024)
Supplement: S4 Table — (DOCX) [file pone.0180024.s004.docx]

*Suppl. Table 4*

*Preoperative Patient Characteristics for Each Treatment Group in SF12 Group*

|  | TSFS (n=14) | MFC (n=8) | p Value |
| --- | --- | --- | --- |
| Age at Operation | 74.5 (40 - 83), 71.9±10.5^z^ | 70.5 (54 - 84), 68^z^ | 0.516^a^ |
| Male Gender | 12 (85.7%) | 5 (62.5) | 0.286^b^ |
| EuroScore additiv | 8 (3-12), 7.9±3.0^z^ | 9 (5-15), 9.1±3.1^z^ | 0.482^a^ |
| EuroScore logitic | 9.59 (2.56 - 27.4), 12.3±8.7^z^ | 12.5 (3.44 - 52.25), 17.0±15.4^z^ | 0.482^a^ |
| LVEF, % | 55.5 (30 - 89), 52.1±15.8^z^ | 60 (20 - 80), 50.5±22.4^z^ | 0.868^a^ |
| Body Mass Index, kg/m^2^ | 30 (25 - 45), 31.5±6.2^z^ | 32 (21 -44), 31±6.9^z^ | 0.973^a^ |
| Diabetes mellitus | 5 (35.7%) | 2 (25.0%) | 1.0^b^ |
| Chronic Obstructive Pulmonal Disease | 2 (14.3%) | 2 (25.0%) | 0.60^b^ |
| Hypertension | 11 (78.6%) | 8 (100%) | 0.273^b^ |
| Perpiheral Vascular Disease | 3 (21.4) | 1 (12.5%) | 1.0^b^ |
| Active smoker or stopped <3 months | 2 (14.3) | 1 (12.5%) | 1.0^b^ |
| Steroids | 2 (14.3) | 1 (12.5%) | 1.0^b^ |
| HO Stroke/Transient Ischemic Attack | 3 (21.4) | 1 (12.5%) | 1.0^b^ |
| HO Myocardial Infarction | 7 (50.0) | 7 (87.5%) | 0.167^b^ |
| Osteoporosis | 2 (14.3) | 0 (0%) | 0.515^b^ |
| HO Cardiac Operation | 0 (0.00) | 2 (25%) | 0.121^b^ |
| *Data are presented as Median and Range with ^z^Mean or absolute value and percentage (%). HO = History of.  ^a^Calculated by Mann-Whitney U test. ^b^Calculated by two-tailed Fisher’s exact test. | | | |
