# Supplementary material for: Therapy options in deep sternal wound infection: Sternal plating versus muscle flap
Source: PLoS One. 2017 Jun 30;12(6):e0180024. doi: 10.1371/journal.pone.0180024 (PMC5493354; doi:10.1371/journal.pone.0180024)
Supplement: S5 Table — (DOCX) [file pone.0180024.s005.docx]

*Suppl. Table 5*

*Intra- and postoperative Patient Data in SF-12 Group*

|  | TSFS (n=14) | MCF(n=8) | p |
| --- | --- | --- | --- |
| Postoperative ICU Stay (d) | 3.5 (2 - 20), 5.9±6.0^z^ | 4 (2 - 69), 13.5±22.9^z^ | 0.525^a^ |
| Cardiac Operation Time (min) | 229.5 (134 - 462), 241.93±79.7^z^ | 265 (155 - 400), 265.6±71.1^z^ | 0.330^a^ |
| Emergency Operation | 4 (28.6%) | 1 (12.5%) | 0.613^b^ |
| Pneumonia | 6 (42.9%) | 3 (37.5%) | 1.0^b^ |
| PostOP Delir | 8 (57.1%) | 3 (37.5%) | 0.659^b^ |
| DSWI | 14 (100%) | 8 (100.00) | 1.0^b^ |
| PostOP Bleeding | 1 (7.1%) | 2 (25%) | 0.527^b^ |
| Seroma Formation | 6 (42.9%) | 2 (25%) | 0.649^b^ |
| Tracheotomy | 0 (0%) | 0 (0%) | 1.0^b^ |
| VAC usage | 14 (100%) | 8 (100%) | 0.273^b^ |
| Number of VAC changes | 3 (1 - 5), 2.7±1.3^z^ | 4 (3 - 5), 4±0.8^z^ | 0.021^a^ |
| Sternum Revision | 2 (14.3%) | 7 (87.5%) | 0.001^b^ |
| Reanimation | 1 (7.1%) | 3 (37.5%) | 0.117^b^ |

*Data are presented as Median and Range with ^z^Mean or absolute value and percentage (%). HO = History of. ICU = Intensive care unit.

^a^Calculated by Mann-Whitney U test. ^b^Calculated by two-tailed Fisher’s exact test.
