# Supplementary material for: Therapy options in deep sternal wound infection: Sternal plating versus muscle flap
Source: PLoS One. 2017 Jun 30;12(6):e0180024. doi: 10.1371/journal.pone.0180024 (PMC5493354; doi:10.1371/journal.pone.0180024)
Supplement: S6 Table — (DOCX) [file pone.0180024.s006.docx]

*Suppl.Table 6*

*Patient Data after Definite Therapy in SF-12 group*

|  | TSFS (n=14) | MFC (n=8) | p |
| --- | --- | --- | --- |
| Prolonged ICU Stay >2d, Mean Stay at ICU (d) | 1 (7.1%) | 1 (12.5%) | 1.0^b^ |
| Post Op ICU Stay (d) | 0 (0 - 14), 1.0±3.5^z^ | 0 (0 - 3), 0.88±1.3^z^ | 0.264^a^ |
| Median Operation time (min) | 122 (98 - 180), 134.5±28.4^z^ | 150 (60 - 282), 160.25±85.1^z^ | 0.431^a^ |
| Time Difference (d)Heart Surgery to Final Reconstruction | 21.0 (7-37), 23.0±9.0^z^ | 39.5 (22 - 50), 37.8±11.1^z^ | 0.009^a^ |
| Days to Discharge | 11.5 (4 - 91), 17.5±21.7^z^ | 27.5 (8 - 82), 33.88±26.4^z^ | 0.043^a^ |
| Seroma Formation | 3 (21.4%) | 2 (25%) | 1.0^b^ |
| Post Op Bleeding | 1 (7.1%) | 1 (12.5%) | 1.0^b^ |
| No. of Post Op Interventions | 0 (0 – 1), 0.07±0.27^z^ | 1.5 (0 - 3), 1.13±1.4^z^ | 0.018^a^ |

*Data are presented as Median and Range with ^z^Mean or absolute value and percentage (%). HO = History of. ICU = Intensive care unit.

^a^Calculated by Mann-Whitney U test. ^b^Calculated by two-tailed Fisher’s exact test.
